# Correction: Regioselective synthesis of aza-saccharins *via* anionic [1,4] Fries-type rearrangement of aryl sulfonimidoyl fluorides

**DOI:** 10.1039/d6sc90113a

**Published:** 2026-05-27

**Authors:** Mario Leypold, Lorenzo Poli, Max Earl, Okky D. Putra, Karolina Kwapien, Richard J. Lewis, John J. Murphy, Marta Passamonti, Lena M. von Sydow, Victor Spelling, Ioannis Asproudis, Malvika Sardana, Claudia Gatti, Hikaru Seki, Thomas Lemaitre, Radvile Juskaite, Ranganath Gopalakrishnan, Stuart J. Francis, Cristina Gardelli, Per-Ola Norrby, Werngard Czechtizky

**Affiliations:** a Medicinal Chemistry, Research and Early Development, Respiratory and Immunology, BioPharmaceuticals R&D AstraZeneca Pepparedsleden 1 43183 Mölndal Sweden mario.leypold@astrazeneca.com; b Early Product Development and Manufacturing, Pharmaceutical Sciences, R&D AstraZeneca Pepparedsleden 1 43183 Mölndal Sweden; c Early Chemical Development, Pharmaceutical Sciences, R&D AstraZeneca Pepparedsleden 1 43183 Mölndal Sweden; d Early Chemical Development, Pharmaceutical Sciences, R&D AstraZeneca Macclesfield SK10 2NA UK; e Predictive Science, Digital and Automation, Pharmaceutical Sciences, R&D AstraZeneca Pepparedsleden 1 43183 Mölndal Sweden

## Abstract

Correction for ‘Regioselective synthesis of aza-saccharins *via* anionic [1,4] Fries-type rearrangement of aryl sulfonimidoyl fluorides’ by Mario Leypold *et al.*, *Chem. Sci.*, 2026, https://doi.org/10.1039/d6sc00432f.

The authors regret that acetylated sulfonimidoyl fluoride **7a** was depicted incorrectly in Scheme 4A of the original article and that “acyl chloride” in the caption of Scheme 4 should read “acetyl chloride”. The corrected version of Scheme 4 is displayed below. Acetylated sulfonimidoyl fluoride **7a** is formed directly from the aryl sulfonimidoyl fluoridate anion **6a** upon trapping with acetyl chloride and should therefore feature a *tert*-butyl ester group in the *ortho*-position relative to the sulfonimidoyl fluoride moiety, rather than the methyl ester group shown in the original article. The corrected structure is consistent with that provided in the original version of the supplementary information (SI).

**Scheme 4:**
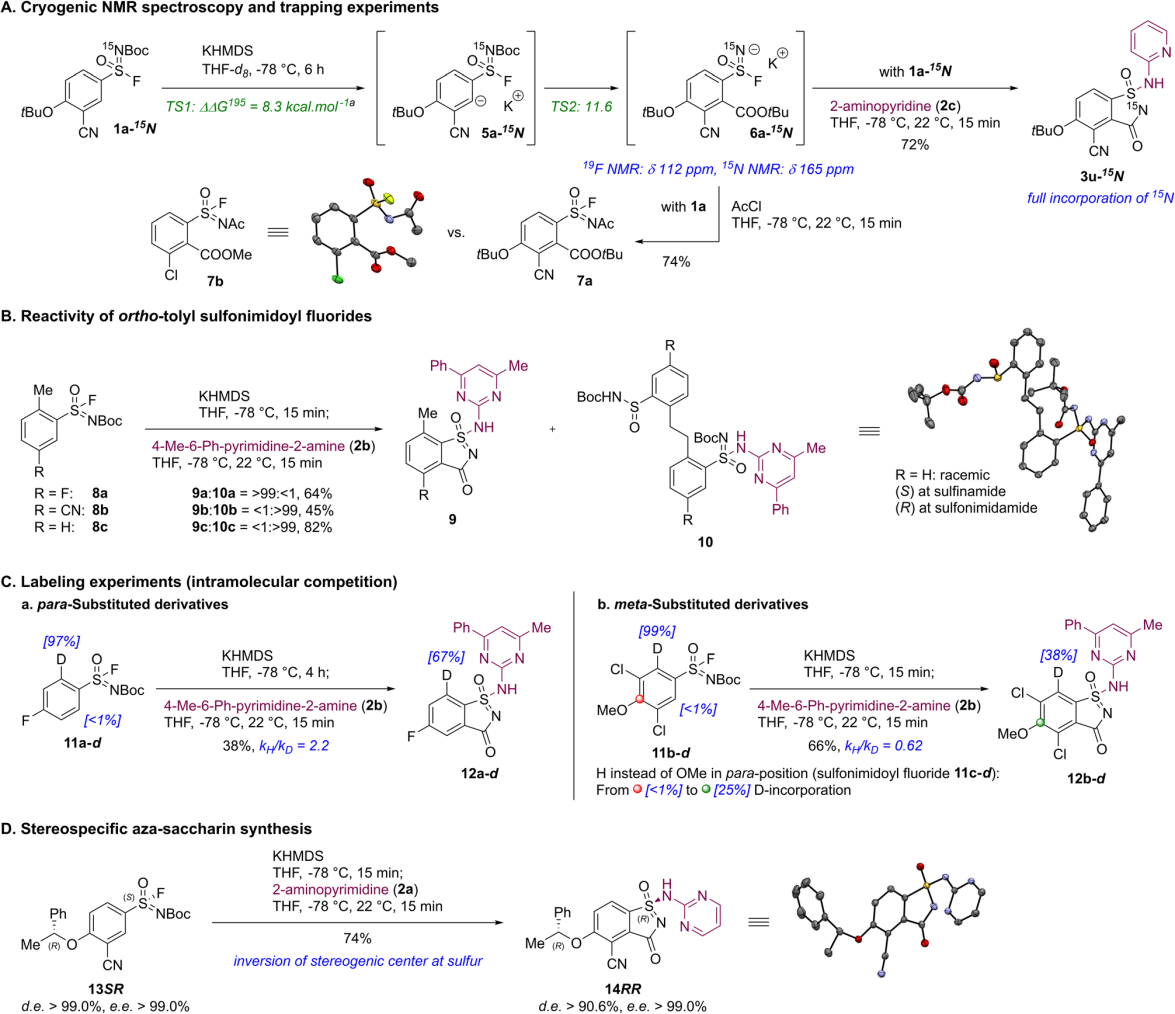
Summary of mechanistic experiments for the formation of aza-saccharins. Reaction conditions: sulfonimidoyl fluoride (1 equiv.), KHMDS (2.00 equiv.), THF, −78 °C; 2-aminopyridine (**2c**, 1.20 equiv.), 4-Me-6-Ph-pyrimidine-2-amine (**2b**, 1.20 equiv.) or acetyl chloride (6.00 equiv.), THF, −78 °C, 22 °C, 15 min. *^a^*Reaction profile for the KPA of unlabeled sulfonimidoyl fluoride **1a** was computed at the M06-2X/def2-TZVP level of theory with implicit solvent model (COSMO) for the treatment of THF. The relative Gibbs free energies ΔΔ*G*^195^ are calculated at 195 K. Values correspond to isolated yields. Displacement ellipsoids are drawn at the 30% and 50% probability level. Hydrogen atoms and minor parts are omitted for clarity.

The Royal Society of Chemistry apologises for these errors and any consequent inconvenience to authors and readers.

